# Symmetry-Protected Moiré Band Engineering and Enhanced Electron–Phonon Coupling in Xe/Bi_2_Se_3_ Superlattices: Path to Topological Superconductivity

**DOI:** 10.1021/acsnano.5c20111

**Published:** 2026-04-09

**Authors:** Asish K. Kundu, Ilya I. Klimovskikh, Alexei V. Fedorov, Elio Vescovo, Genda D. Gu, Tonica Valla

**Affiliations:** † National Synchrotron Light Source II, 8099 Brookhaven National Laboratory, Upton, New York 11973, United States; ‡ 226245 Donostia International Physics Center, Donostia-San Sebastian 20018, Spain; § Advanced Light Source, Lawrence Berkeley National Laboratory, Berkeley, California 94720, United States; ∥ Condensed Matter Physics and Materials Science Department, 8099 Brookhaven National Laboratory, Upton, New York 11973, United States; ⊥ Institut za fiziku, Bijenička 46, Zagreb HR-10000, Croatia

**Keywords:** moiré superlattices, topological insulators, Bi_2_Se_3_, ARPES, topological surface states, electron−phonon coupling, topological superconductivity

## Abstract

Observation of superconductivity, magnetism, and correlated insulating phases driven by the moiré potential in twisted graphene bilayer has opened the exciting new field of “twistronics”. Even richer physics is expected if moiré superlattice could be generated on topological insulators; however, until now, experimental studies have been scarce. Here, we demonstrate topological moirés generated by adsorbing a monolayer of noble gas on a topological insulator. By angle-resolved photoemission spectroscopy, we show that the moiré potential replicates the topological surface state and affects it in a way fundamentally different from the trivial states. Replicated Dirac cones generally avoid crossings, except at the time-reversal invariant momenta that remain gapless. This creates van Hove singularities at the moiré Brillouin zone corners, providing the mechanism of enhancing correlations. Indeed, we observe a strong enhancement of the electron–phonon coupling strength that, if properly tuned, might lead to topological superconductivity and Majorana Fermions.

## Graphical abstract

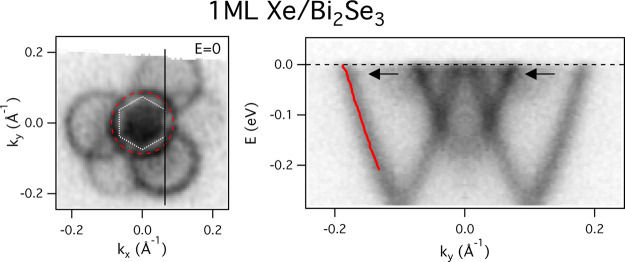

## Introduction

Moiré superlattices have emerged as a powerful tool for engineering novel electronic and topological phases in condensed matter systems. By introducing long-range periodic modulations through stacking of two-dimensional (2D) materials with a slight twist or lattice mismatch, moiré superstructure arises with the periodicity $a_{M}\approx a/\sqrt{\delta^{2}+\theta^{2}}$ . Here, δ = (*a* – *a′*)/*a* is the lattice mismatch and θ is the twist angle between the two constituents of a moiré. Moiré superstructures enable unprecedented control over the electronic band structure and its filling, leading to correlated insulating states, superconductivity, and topological phenomena.^1,2^

In twisted bilayer graphene (TBG), moiré potential replicates the Dirac cone and opens minigaps, leading to flat bands around the “magic” twist angle, resulting in correlation induced phases such as unconventional superconductivity, Mott and fractional chern insulating phases and quantum anomalous Hall effect.^1–8^ In transition metal dichalcogenides (TMD), moiré potential has been reported to result in Wigner crystals and Hubbard physics.^9–13^

Although moiré physics has been extensively explored in trivial systems such as TBG and TMD heterostructures, its implications for topological surface states of three-dimensional (3D) topological insulators (TIs) remained largely unexplored. Theoretical considerations predict that a fundamental distinction between moiré superlattices on TI surfaces and those in trivial materials lies in topological protection of the Dirac surface states.^14–16^ Unlike in TBG, the surface states of TIs are constrained by time-reversal symmetry (TRS) and spin-momentum locking^17–21^ and the nature of Umklapp gaps is expected to be fundamentally different. If the TRS is preserved, avoided crossings and energy gaps should appear generically at the moiré-induced band intersections, except at the time-reversal-invariant momenta (TRIM), i.e., *M′* points of the moiré Brillouin zone (BZ). This should result in van Hove singularities (vHSs) at *K′* points and renormalization of moiré bands, leading to enhanced correlations, fractionalized excitations, and topological superconductivity.^14,15^ Experimentally, however, the moiré superlattices on surfaces of TIs have rarely been realized due to the fabrication challenges related to the 3D TI stacking.^22–24^ One exception is the recent moiré generation by monolayers of magnetic transition metal dihalides on surfaces of TIs,^25^ where the results were affected by the magnetism of the dihalide.^25^

Here, we report the successful realization of nonmagnetic topological moiré superlattices by adsorbing xenon monolayers on 3D TIs, Bi_2_Se_3_, and Bi_2_Te_3_. Using angle-resolved photoemission spectroscopy (ARPES), we investigate the impact of moiré superlattice potential on the topological surface state, focusing on the gap formation at the moiré-induced intersections. We find that the avoided crossings generally form, except at *M′* points of the moiré BZ that remain gapless, in accord with predictions.^14,15^ The maximal hybridization gap occurs at the *K′* points, 2Δ ≈ 27 meV, which thus become van Hove singularities, enhancing the correlations. Indeed, we observe a strong enhancement of the electron–phonon coupling (EPC) in the Xe/Bi_2_Se_3_ moiré, which, with additional tuning, represents a promising path toward topological superconductivity in these systems.^14,15^

## Results and Discussion

Figure 1 shows the wide energy range ARPES spectra of approximately one monolayer (1 ML) of Xe adsorbed on Bi_2_Se_3_ (Figure 1a–c) and on Bi_2_Te_3_ (Figure 1d–f) recorded at *h*ν = 50 eV photons. The zoomed-in regions, corresponding to the Xe 5*p* bands and the topological surface state (TSS), are shown in the (c, e) and (b, d) panels, respectively. The Bi_2_Se_3_ and Bi_2_Te_3_ single crystals were mounted on the same plate, cleaved at the same time and exposed to the same dose of Xe. The Xe 5p states form sharp bands with significant dispersion, indicating the high structural quality of the adsorbed layer. However, on Bi_2_Se_3_, the Xe bands display somewhat wider bandwidth and more complex structure than on Bi_2_Te_3_. This is probably related to the larger mismatch of the Xe lattice with Bi_2_Se_3_ and the creation of an incommensurate moiré structure. The effects of moiré superlattice on the topological surface state are not clearly visible at this photon energy. The intensity of the moiré-induced replicas of the main TSS cones is negligible, and only the originals can be seen, although attenuated by the Xe monolayer. This changes drastically when the spectra are taken at lower photon energies (18–30 eV range) where the replicas become clearly visible.

**Figure 1:**
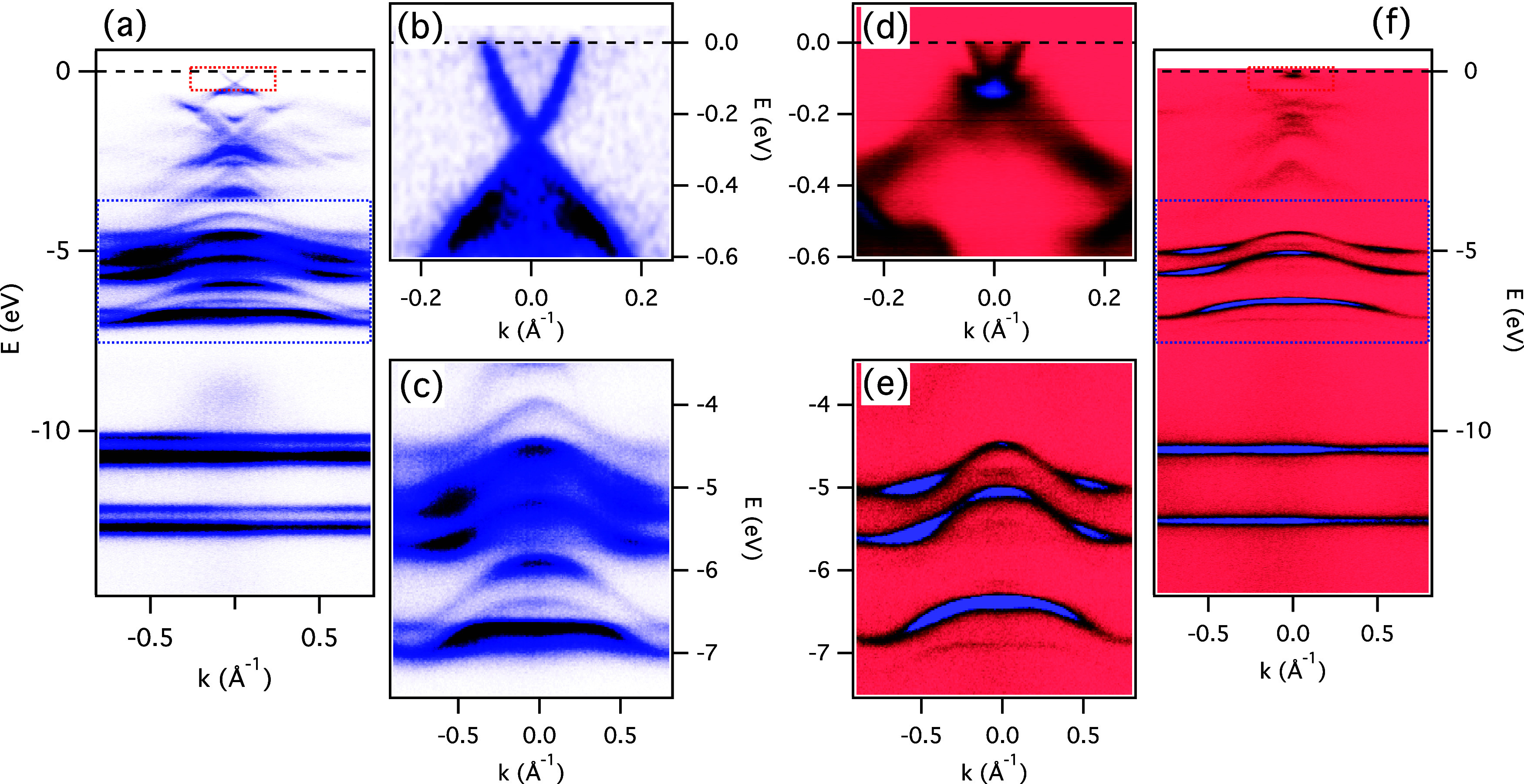
Xe on topological insulators. (a) Wide energy range ARPES spectrum of a monolayer of Xe adsorbed on Bi_2_Se_3_, with the marked boxes representing the TSS (red) and Xe 5p bands (blue), shown in close-ups in (b) and (c), respectively. The corresponding spectra for Bi_2_Te_3_ are shown in (d–f). All the spectra were recorded at *h*ν = 50 eV, at *T* = 15 K.

Figure 2 shows the low-energy electronic structure of the same samples from Figure 1, now taken at *h*ν = 22 eV. The moiré induced replicas are now clearly visible for the Xe/Bi_2_Se_3_ system. It is interesting that the TSS cone is replicated with the intensity similar to the original one, whereas the replicas of the quantum well states (QWSs) originating from the conduction band^29^ are much weaker than the original. The bulk valence states do not seem to be replicated at all. This is likely a consequence of the varying degree of localization of these states to the interface with Xe, where the moiré potential acts and affects the electrons in these states differently.

**Figure 2:**
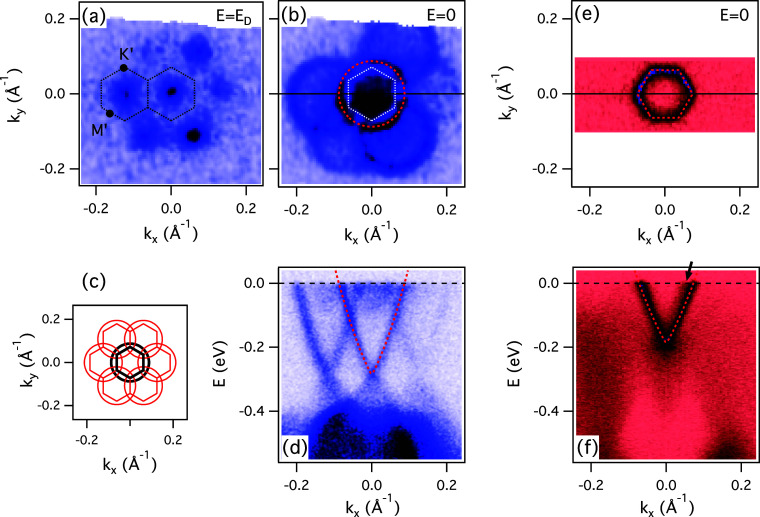
Moiré electronic structure of the Xe monolayer on TIs. (a) Constant energy ARPES map of Xe/Bi_2_Se_3_ at *E* = *E*_D_ = −0.288 eV, showing the Dirac points of the original and replicated TSSs. The moiré mini-BZs are indicated (dashed hexagons). (b) Same at *E* = 0 (Fermi surface). The red dashed circle represents the Fermi surface of the original TSS fitted by a second order polynomial, as described in the SI. Dashed white hexagon represents the 1st moiré BZ. (c) Schematic view of the original (black) and replicated Fermi surfaces (red) and moiré BZs. (d) *E* vs *k* dispersion along the Γ*′M′*Γ*′* line indicated in (b). The corresponding Fermi surface and dispersion for Bi_2_Te_3_ are shown in (e) and (f), respectively. All the spectra were recorded at *h*ν = 22 eV, at *T* = 15 K.

Based on the displacement of the replicas in *k* space, we can determine both the moiré period in the real space *a*_m_ ≈ 60 Å and the Xe lattice parameter, *a*_Xe_ ≈ 4.43 Å for the Xe/Bi_2_Se_3_ case, using the published lattice constant for Bi_2_Se_3_ (see Table 1). The obtained Xe–Xe interatomic distance agrees very well with that in Xe bulk crystal,^28^ very close to the Bi_2_Te_3_ lattice constant, thus making the Xe/Bi_2_Te_3_ interface either perfectly matched, or with a very long moiré period. As a consequence, the replicas of the Bi_2_Te_3_ surface state (if they exist) are difficult to resolve (Figure 2e,f). If the weak intensity marked by the arrow reflects the replica, then the lower bound on the moiré period is *a*_m_ ∼ 24 nm (Δ*k* ≤ 0.015 Å). We note that neither in Xe/Bi_2_Se_3_ nor in Xe/Bi_2_Te_3_ could we detect the change in moiré period with temperature in the range of 15 to 60 K, contrasting the behavior in graphene and TiSe_2_, where a significant expansion of adsorbed Xe was observed over the same temperature range.^30,31^ If correct, the thermal expansion coefficients from refs 30 and 31 would be roughly 30 times larger than in the bulk Xe.^28^

**Table 1:** Lattice Parameters for Bi_2_Se_3_,^26^ Bi_2_Te_3_,^27^ Xe,^28^ Xe/Bi_2_Se_3_, and Xe/Bi_2_Te_3_ ^a^

material	*a* (Å)	*c* (Å)	*T* (K)
Bi_2_Se_3_	4.126	28.48	10
Bi_2_Te_3_	4.383	30.49	4
Xe	6.2 (4.38)	6.2	75
Xe/Bi_2_Se_3_	60		15
Xe/Bi_2_Te_3_	>240		15

a Xe crystallizes in the fcc cubic structure with the interatomic distance given in parentheses. *T* indicates the temperature at which the parameters are obtained.

It is interesting that the replicas in Xe/Bi_2_Se_3_ shown in Figure 2d do not display any hybridization, i.e., their crossings are gapless. This is drastically different from graphene moiré superlattices where the crossings are always avoided (gaped). In principle, the gapless crossings could be taken as evidence that the replicas might be the diffraction images of outgoing photoelectrons on the superlattice formed by Xe and the sample. If that is indeed the case, the initial states do not necessarily feel the long-period potential and no gaps need to open at the intersections. A similar situation was long thought to exist in a cuprate high-temperature superconductor Bi_2_Sr_2_CaCu_2_O_8+δ_ where the mismatch between the constituent Bi–O and Cu–O planes results in a 1D structural supermodulation, producing replicas of the Fermi surface visible in the electronic structure.^32^ However, since no reconstruction was observed, these replicas were thought to be a diffraction effect with no supermodulation influencing the electronic states in the Cu–O planes. Only recently, after improvements in resolution, has it become possible to detect the hybridization gaps and uncover the direct effect of the modulation potential on the states in the Cu–O planes.^33^ After that, exciting effects on the superconducting gap have been observed, allowing the sign of the superconducting order parameter to be detected in ARPES.^33,34^

By this analogy, one could say that in the present case, too, the replicas might be diffraction images of the main Dirac cone. Furthermore, the argument against diffraction, presented in ref 31 does not work, as the diffraction should involve reciprocal wave-vector of the moiré, and not of the Xe lattice.

One indication that in our case the replicas are not diffraction images is the fact that the TSS, the conduction band-derived QWSs and the bulk valence states are replicated with very different strengths. This suggests that the replication efficiency follows the degree of localization of each state to the interface, where the moiré potential acts.

However, crucial proof that the moiré potential does affect the topological states and that it does so in a fundamentally different way than in topologically trivial systems is shown in Figure 3. We now focus solely on the Xe/Bi_2_Se_3_ case, where the moiré period is better suited to resolve the small hybridization effects. While the Fermi surface does not show obvious signs of reconstruction (panel (a)), and the dispersion along the Γ*′M′*Γ*′* (line 1) does not display avoided crossings (panel (b)), the dispersions corresponding to lines 2 and 3 from panel (a) indicate that the hybridization can now be observed at the intersections along these lines (panels (c, d, e, f, g)). We note that line 2 runs along the Γ*′K′M′*Γ*′* line where three cones (the original and two replicas) intersect at each *K′* point of the moiré BZ. Although the spectral intensity is not reduced at the intersections, the upper portion of the dispersion is clearly displaced in energy from the continuation of the lower portion near the intersection, with the apparent “vertical” dispersion in between the two parts. This is better visible in the close-up in the panel (g) and in (d^2^/(d*k*)^2^) of the spectral intensity plot (panel (f)). The “vertical” dispersion is usually an indication of a spectral gap, often observed when the gap is not “clean”, but instead becomes filled due to disorder, or when it is smaller than the spectral width of the states forming it.^35,36^ The gap magnitude is extracted using the procedure described in the SI, Figures S1 and S2.

**Figure 3:**
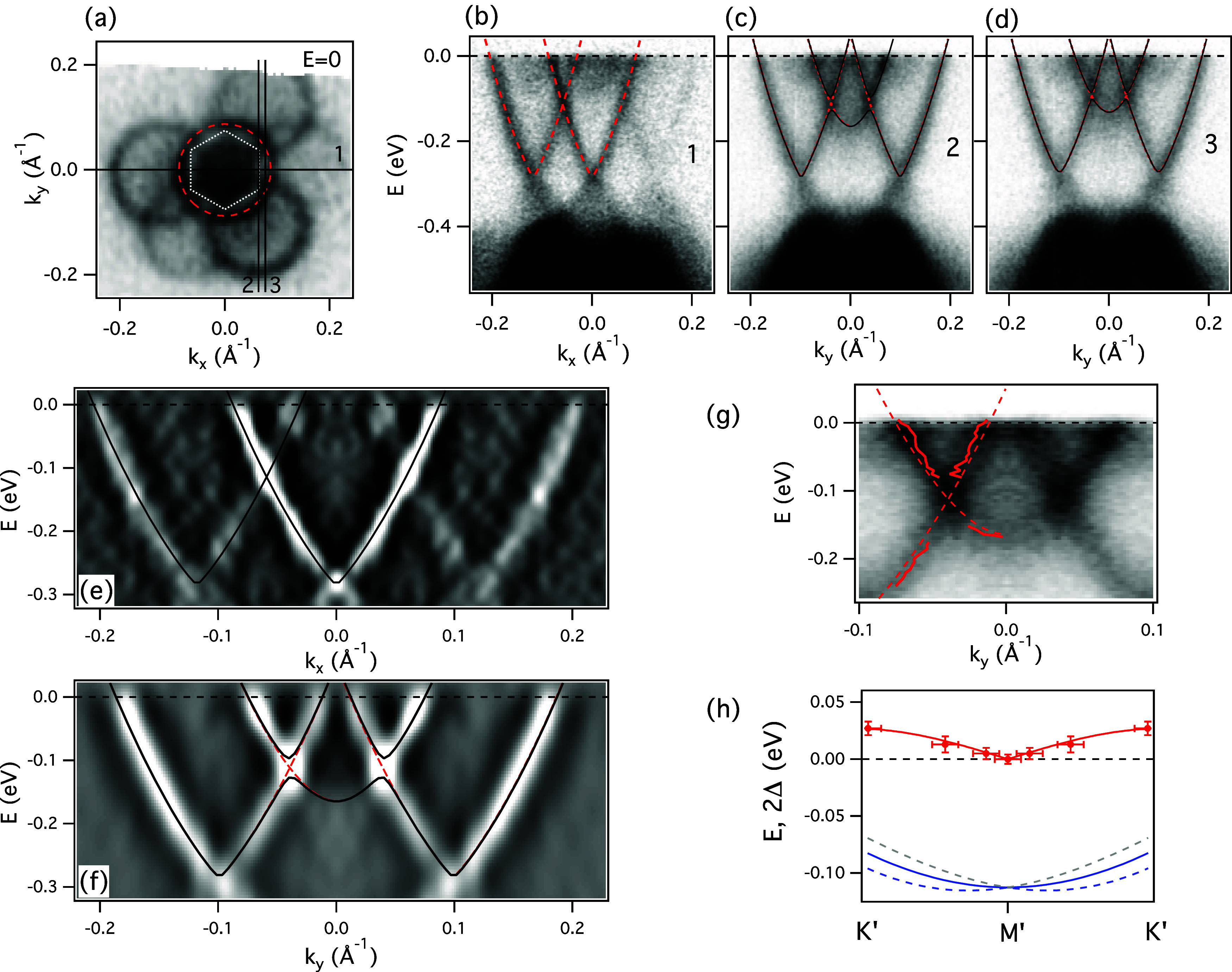
Moiré mini-gaps for Xe/Bi_2_Se_3_. (a) Constant energy ARPES map at *E* = 0 ± 3 meV from Figure 2b with the momentum lines probed in (b–g) indicated by the numbered solid lines. (b–d) *E* vs *k* dispersions along the corresponding lines indicated in (a). The red dashed curves represent dispersions of the fitted original TSS and replicas displaced by the moiré reciprocal wave vector without any hybridization. The solid black curves include hybridization gaps at the crossings that best describe the measured dispersions. (e, f) Close-up of the spectra in (b) and (c), showing the second derivative of intensity along the momentum direction, (d^2^/(d*k*)^2^). (g) Close-up of the raw data from (c). Solid curves represent experimental dispersions obtained by fitting the MDCs with Lorentzian peaks. (h) Moiré gap and its dispersion along the moiré zone boundary in Xe/Bi_2_Se_3_. Red symbols and the red curve represent the measured gap magnitude and its assumed sine dependence along the *K* *′M* *′K* *′* line. The solid blue curve represents the dispersion of gapless intersections, while the dashed curves indicate the hybridization gap edges. All the spectra were recorded at *h*ν = 22 eV, at *T* = 15 K.

To account for the observed shift between the upper and lower parts of the measured dispersions, and to illustrate that these dispersions do require hybridization gaps, we invoke the simple hybridization model where the gap magnitudes are added by hand using the procedure described in the SI: $E_{\pm }(k)=(E_{1}(k)+E_{2}(k))/2$ ± $\sqrt{(E_{1}(k)-E_{2}(k){)}^{2}+4\Delta (k{)}^{2}}$ . *E*_1_(*k*) and *E*_2_(*k*) represent the two noninteracting intersecting cones obtained by fitting the pristine TSS cone by the 2nd order polynomial, and Δ­(*k*) is the gap, or the interaction between the hybridizing states. The interaction is strongest when the intersection is close to the *K′* point and vanishes at the *M′* point of the moiré BZ. The maximal gap at *K′* is estimated to be 2Δ ∼ 27 meV (see the SI text and Figure S2b for more details).

This is summarized in Figure 3h where we plot the magnitude of the gap and the position of intersections along the moiré zone boundary, as described in the SI and Figures S1 and S2. The gap splits the noninteracting intersecting line and a highly anisotropic mini-cone centered at *M′* point is formed, with high order vHSs at *K′* points, in accordance with theoretical predictions.^14^ The position of these vHSs relative to the Fermi level depends on the size of the moiré lattice, details of the original cone and on its filling.

This brings us to the second important result of this study: enhanced electronic correlations. It is well established that due to the extremely weak EPC within the TSS cone, superconductivity on the pristine surface is highly unlikely to occur.^38–40^ However, the gaps and the resulting higher order vHSs observed here could dramatically enhance correlations in topological moiré superlattices, including the EPC. In fact, recent theoretical analysis suggests that the pairing correlations would dominate, even within the conventional EPC picture, potentially leading to topological superconductivity.^14,15^ In order for that to happen, the vHSs should be brought relatively close to the Fermi level, so that the electronic density of states is increased within the phonon range. This could be done either by doping, or by tuning the moiré period (by a choice of the adsorbed layer or the TI substrate). Also, in the present case of Bi_2_Se_3_, the second crossing along the Γ*′K′M′K′*Γ*′* line occurs at *M′* point just above the Fermi level. A small increase in the moiré period or in the filling could fine-tune this crossing to the Fermi level, which is predicted to additionally enhance the coupling by renormalizing the state’s velocity at this TRS protected crossing.^15^

The correlation enhancement seems to be already taking place in our current Xe/Bi_2_Se_3_ system as the EPC seems to be already visibly enhanced relative to the pristine case of Bi_2_Se_3_.^39,40^ Figure 4 illustrates the change in the measured EPC as Xe is desorbed after the study of the Xe/Bi_2_Se_3_ moiré superalattice. As Xe is desorbed, the pristine TSS is slightly more filled, but the faint anomaly in the state’s dispersion (“kink”) that was visible when Xe was adsorbed, now disappears. The “kink” in dispersion at these low-energies is usually caused by the EPC,^37^ an interaction responsible for superconductivity in conventional superconductors. The clear presence of the “kink” in Figure 4a is a strong evidence that the EPC is enhanced in the moiré superlattice. The quantitative analysis is described in more details in the SI, and Figure S3, with the main results summarized here in Figure 4c, showing the ReΣ­(*E*) for the two cases. The low-energy slope of ReΣ (indicated by the straight lines in (d)) represents the EPC strength, λ. For the pristine sample, the coupling is very weak, λ ≈ 0.1 ± 0.05, in agreement with previous studies,^39,40^ but for the Xe/Bi_2_Se_3_ moiré, it is significantly enhanced, λ ≈ 0.34 ± 0.03. According to ref 14, this λ would have already resulted in *T*_c_ of ∼ 10 K. However, the problem is that proximity of metallic bulk of Bi_2_Se_3_ would almost certainly reduce surface superconductivity, or kill it completely. Also, the vHSs are still far from the Fermi level and these theoretical considerations should not apply. Nevertheless, by fine-tuning of our system, for example by surface doping, the EPC might be enhanced further, and eventually the topological superconductivity should be observed. We also point out that adsorbed Xe brings its own low-frequency phonons that might enhance the coupling irrespective of moiré effects. However, this is an unlikely scenario here, as the peak in ReΣ, that reflects the energy of the involved phonons, would definitely exclude xenon’s due to their very low-energy.^41^ The peak matches the energy of the optical Bi_2_Se_3_ phonons,^42^ while the *k*-dependence of the coupling strength (Figure S3) suggests that the phonons with small *q* are involved in the coupling. This additionally reaffirms that the enhanced density of electronic states due to moiré formation is the origin of the enhanced coupling. We hope that these factors and some open questions related to possible superconductivity in this and similar systems should be addressed in future theoretical and experimental studies.

**Figure 4:**
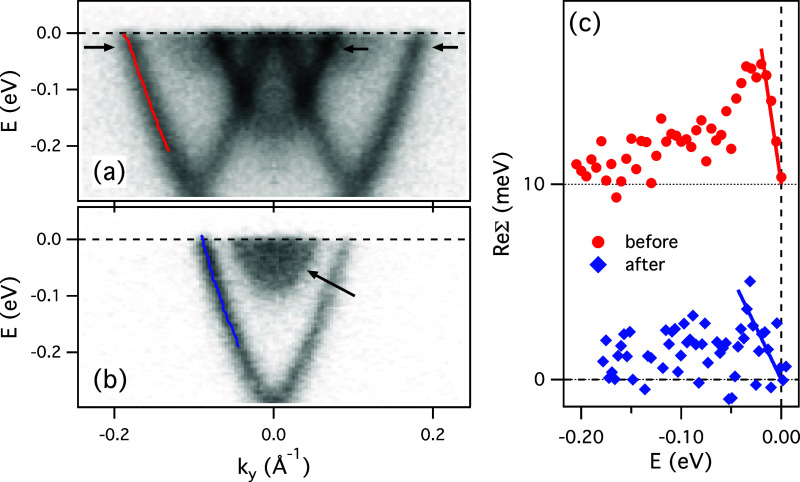
Electron–phonon coupling in the Xe/Bi_2_Se_3_ moiré. (a) Dispersion of the intersecting TSS cones in Xe/Bi_2_Se_3_ from Figure 3. The red curve represents the peak positions of the momentum distribution curves (MDC).^37^ The arrows point to the anomalies in dispersions. (b) Same Bi_2_Se_3_ sample after Xe was completely desorbed. The blue curve represents the MDC determined dispersion. The arrow points to the conduction band QWSs. (c) Experimentally determined ReΣ for the two cases from (a) and (b) as explained in the SI. The solid lines represent the linear fits to the low-energy part of ReΣ before (red) and after (blue) desorbing Xe. The red symbols and line are shifted up by 10 meV for clarity.

## Conclusions

In summary, we have generated homogeneous topological moiré superlattices by adsorbing Xe on surfaces of 3D TIs. The electronic structure shows the replicated TSS Dirac bands with the gapless crossing at the *M′* point and (gapped) avoided crossings elsewhere on the moiré BZ boundary. This results in anisotropic topological Dirac cones centered at *M′* points with vHS at *K′* points and enhanced density of states, in agreement with the theoretical predictions.^14,15^ We observe a significant enhancement of EPC in the Xe/Bi_2_Se_3_ moiré relative to the pristine Bi_2_Se_3_ case. With the additional tuning, this could be optimized, potentially leading to topological superconductivity and Majorana Fermions.

It is important to stress that our method of moiré patterning could also be applied to many other systems, including the trivial, superconducting and magnetic ones, with the potential to become a cleaner alternative to “twistronics”. In contrast to the top-bottom approach of the latter, based on manipulations of micron-sized exfoliated flakes, our study demonstrates a bottom-top approach with the possibility of synthesis on a wafer scale. Our method always results in high quality moirés, where the replicated states are equally sharp as the starting ones. We also note that the parameter space can be significantly expanded if magnetism is added to the game, by adsorbing magnetic layers and breaking the TRS in the moiré lattice.^16,25,43^ Our recent studies involving the layers of van der Waals magnets grown on TIs show that the moiré gaps can be manipulated by magnetism.^25^ We hope that our results will stimulate further studies in this exciting new area of topological moiré superlattices.

## Methods

The single crystals of Bi_2_Se_3_ and Bi_2_Te_3_ were grown from high-purity (99.9999%) elements by a modified floating zone method, where the Se­(Te)-rich material was used in the melting zone. The crystal growth rate was controlled at 0.5 mm/h.

The ARPES experiments were performed at the Electron Spectro-Microscopy (ESM) 21-ID-1 beamline of the National Synchrotron Light Source II and at BL-10.0.1.2 of the Advanced Light Source. Both beamlines are equipped with the Scienta DA30 electron analyzers, with base pressure ∼2 × 10^–11^ mbar. The total energy resolution (at *h*ν = 22 eV) was ∼6 and ∼12 meV at ESM and BL-10.0.1.2, respectively. The angular resolution was ∼0.15° and ∼0.3° along the slit and perpendicular to it, respectively, in both facilities.

Approximately 3 L of Xe (5 × 10^–9^ mb, for 10 min) was adsorbed at ∼50 K to ensure the ordered growth of the first layer and prevent the growth of the second layer. The samples were then cooled to the base *T* (≈15 K) and measured in ARPES. The Xe monolayer would begin to desorb around 55–60 K and would quickly completely desorb around 70 K from both Bi_2_Se_3_ and Bi_2_Te_3_. After desorption, the TSSs on both materials would return to preadsorption state, albeit sometimes with a small downshift of the Dirac point.

## Supplementary Material



## Data Availability

The data that support the findings of this study are available from the corresponding author upon reasonable request.

## Notes

Details on preparation of Xe monolayers, determination of hybridization gaps and the electron–phonon coupling strength, and additional ARPES data (PDF)
