# Supplementary material for: Symmetry-Protected Moiré Band Engineering and Enhanced Electron–Phonon Coupling in Xe/Bi2Se3 Superlattices: Path to Topological Superconductivity
Source: ACS Nano. 2026 Apr 9;20(15):11692–8. doi: 10.1021/acsnano.5c20111 (PMC13104177; doi:10.1021/acsnano.5c20111)
Supplement: Supplementary file 1 [file nn5c20111_si_001.pdf]

Supplementary Information for:

Symmetry-Protected Moiré Band Engineering and Enhanced Electron–Phonon Coupling in Xe/Bi_2Se_3 Superlattices: Path to Topological Superconductivity

Asish K. Kundu,[†] Ilya I. Klimovskikh,[‡] Alexei V. Fedorov,[¶] Elio Vescovo,[†] Genda D. Gu,[§] and Tonica Valla[*,‡,‖]

†*National Synchrotron Light Source II, Brookhaven National Laboratory, Upton, New York 11973, USA*

‡*Donostia International Physics Center, 20018 Donostia-San Sebastian, Spain*

¶*Advanced Light Source, Lawrence Berkeley National Laboratory, Berkeley, California 94720, USA*

§*Condensed Matter Physics and Materials Science Department, Brookhaven National Laboratory, Upton, New York 11973, USA*

‖*Institut za fiziku, Bijenička 46, HR-10000 Zagreb, Croatia*

E-mail: tonica.valla@dipc.org

This document contains:

- Details on the preparation of Xe monolayers
- Details on how the hybridization gaps were determined.
- Details on how the electron-phonon coupling strength was determined.
- Three supplementary figures referenced in the main text.

Preparation of Xe monolayers

Xe is a noble gas and does not make strong chemical bonds, but is strongly polarizable and bonds to the substrates through the van-der-Waals interaction. The low T ($T < 40$ K) adsorption of Xe does not result in well formed moiré - the ARPES in that case shows only the broadened original TSS cone, surrounded by a background of diffuse intensity, whereas the Xe $5p$ states form a broad continuum without well defined bands, indicating a disordered growth. After annealing at slightly higher temperatures ($T \sim 50-55$ K), the diffuse intensity transforms into the moiré replicas, indicating desorption of the thicker layers and ordering of the Xe monolayer. The annealing is stopped when the spectral width of the TSS cones is nearly the same as the one corresponding to the original TSS on a pristine Bi_2Se_3 surface. The same quality of the Xe monolayer can be achieved if Xe is adsorbed at $\sim 50-55$ K preventing the growth of the second layer during the adsorption. The samples were then cooled to the base T (≈ 15 K) and measured in ARPES.

Based on the spectral width of the TSS cones, the sharp and well formed Xe $5p$ bands and the well ordered constant energy contours of ARPES intensity, we can assume that the level of disorder introduced by the Xe monolayer is negligible and that the Xe monolayer is aligned with the substrate without any twist.

Hybridization gaps

Since the width of pristine TSS is of the order of ~ 100 meV, the hybridization gaps at the crossings of replicated TSS cones will not result in reduced spectral intensity inside the gap, when the gaps are smaller than the width of the states. Thus, the gaps are filled, but their existence will still be reflected through the anomaly in dispersion - a vertical jump in the measured dispersion near the intersects. This is how the gap magnitudes are determined - they are estimated from the measured MDC dispersions from the E(k) region near the crossings, but far enough so that the two-peak structure in MDC is still visible and the

fitting is reliable (black spectrum in Fig. S1(f)). The left-hand and right-hand sides of the dispersion are then extrapolated to the point of their intersect using linear fitting to the peak positions (Fig. S1 and S2). We note that near the band's minima, where the MDC fitting is not possible (lines 2 and 3), the band dispersion is extracted by fitting the EDCs, and the 2nd order polynomial is used to fit the peak dispersion. The vertical distance (energy shift) between the upper and lower intersects is taken as the measured gap, $2\Delta(k)$. This gap is then used as an *ad hoc* parameter to model the hybridized dispersions: $E_{\pm}(k) = (E_1(k) + E_2(k))/2 \pm \sqrt{(E_1(k) - E_2(k))^2 + 4\Delta(k)^2}$, where $E_1(k)$ and $E_2(k)$ are two non-ineracting intersecting cones and $\Delta(k)$ is the hybridization gap. We note that this model is taken only as a visual illustration of avoided crossings and it was not used as a model to fit the observed dispersion and the gap.

The error bars for the hybridization gap (Fig. 3(h), main text) come from the uncertainty in determining the upper and lower intersects from the linear fits to the MDC derived dispersions above and below the intersect (Fig. S1 and S2). For the lines 2 and 3 (Fig. S1), the gap and the error bars are similarly determined using the quadratic extrapolation for the intersecting main TSS cone (Fig. S2(b)). The $x-$error bars (Fig. 3(h), main text) come from the uncertainty in determining the exact wave-vector and are approximated by the finite momentum resolution, coming form the finite angular resolution of the electron analyzer.

Electron-phonon coupling

The electron-phonon coupling (EPC) strength is determined in a standard way used in ARPES,[1] from the dispersion anomaly ("kink") observed in the measured spectral function near the Fermi level:

$$\mathrm{A}(E, \mathbf{k}) \propto \frac{\mathrm{Im}\Sigma(E, \mathbf{k})}{[E - \epsilon_{\mathbf{k}} - \mathrm{Re}\Sigma(E, \mathbf{k})]^2 + (\mathrm{I}m\Sigma(E, \mathbf{k}))^2}$$

where $\epsilon_{\mathbf{k}}$ represents the energy of the non-interacting state and $\Sigma(E, \mathbf{k})$ is the quasiparticle self-energy reflecting the EPC. The dispersion anomaly, $\mathrm{Re}\Sigma(E, \mathbf{k})$, is obtained from the difference between the measured, MDC-derived dispersion, and the putative "non-interacting" dispersion, $\epsilon_{\mathbf{k}}$, that the state would have had without the EPC. The low energy slope of $\mathrm{Re}\Sigma(E, \mathbf{k})$ represents the mass enhancement, $\lambda = -[\partial(\mathrm{Re}\Sigma)/\partial E]_0$, that can be obtained by fitting the low energy part of $\mathrm{Re}\Sigma(E, \mathbf{k})$ to a straight line (Fig. S3).

As a first step, the non-interacting dispersion $\epsilon_{\mathbf{k}}$ is determined by fitting the measured dispersion of the TSS cone of the pristine surface to the 2nd order polynomial. Here we note that for Bi_2Se_3 the hexagonal warping term was negligible, unlike in Bi_2Te_3 where it was necessary to properly describe the shape of the TSS cone. The fitting is performed over the wide energy range above the Dirac point, except for the narrow ($\sim 30 - 40$ meV) interval near the Fermi level where EPC acts. The resulting dispersion for Bi_2Se_3, $\epsilon(k_x, k_y) = 12.097(k_x^2 + k_y^2) + 2.2309\sqrt{k_x^2 + k_y^2} - E_D$ is then subtracted from the measured one and the difference is multiplied by the corresponding group velocity of non-interacting cone to obtain $\mathrm{Re}\Sigma(E)$. The same $\epsilon(k_x, k_y)$ is used to determine $\mathrm{Re}\Sigma(E)$ in Xe/Bi_2Se_3 moiré superlattices. From the width (W) of the MDC peaks, we can obtain $\mathrm{Im}\Sigma(E)$, $2\mathrm{Im}\Sigma(E) = W(E)v(E)$, where $v(E)$ is the velocity of non-interacting band. $\mathrm{Re}\Sigma$ and $\mathrm{Im}\Sigma$ are causally related, reassuring that they both are caused by interacting of TSS electrons with phonons and not by some other effect, such as (avoided) crossings with CB QWS.

The EPC constant, λ, is obtained by fitting the low energy part ($E < 20$ meV) of $\mathrm{Re}\Sigma$ with the straight line going through zero at $E = 0$. While for the pristine surface we obtain very weak EPC, $\lambda \sim 0.1 \pm 0.05$, the coupling is significantly stronger when Xe monolayer covers the surface, $\lambda \sim 0.34 \pm 0.03$, for the same point on the Fermi surface. The uncertainties correspond to one standard deviation in the linear fit.

Measurements show measurable variation along the Fermi surface in the Xe/Bi_2Se_3 case. The coupling seems to be maximal for line 3, when the Fermi surface is closest to the vHS at the K' point. This, and high energy of the "kink", $\sim 15 - 25$ meV would imply that the

small momentum Bi_2Se_3 optical modes are responsible for the observed coupling, rather than the very low-energy Xe modes.[2,3] This also implies that the coupling is enhanced precisely because of the moiré formation due to an increase in density of states near the induced vHS at the K' point.

References

1. Valla, T.; Fedorov, A. V.; Johnson, P. D.; Hulbert, S. L. Many-Body Effects in Angle-Resolved Photoemission: Quasiparticle Energy and Lifetime of a Mo(110) Surface State. *Physical Review Letters* **1999**, *83*, 2085–2088.

2. Lurie, N. A.; Shirane, G.; Skalyo, J. Phonon dispersion relations in xenon at 10 K. *Phys. Rev. B* **1974**, *9*, 5300–5306.

3. Boulares, I.; Shi, G.; Kioupakis, E.; Lošťák, P.; Uher, C.; Merlin, R. Surface phonons in the topological insulators Bi2Se_3 and Bi_2Te_3. *Solid State Communications* **2018**, *271*, 1–5.

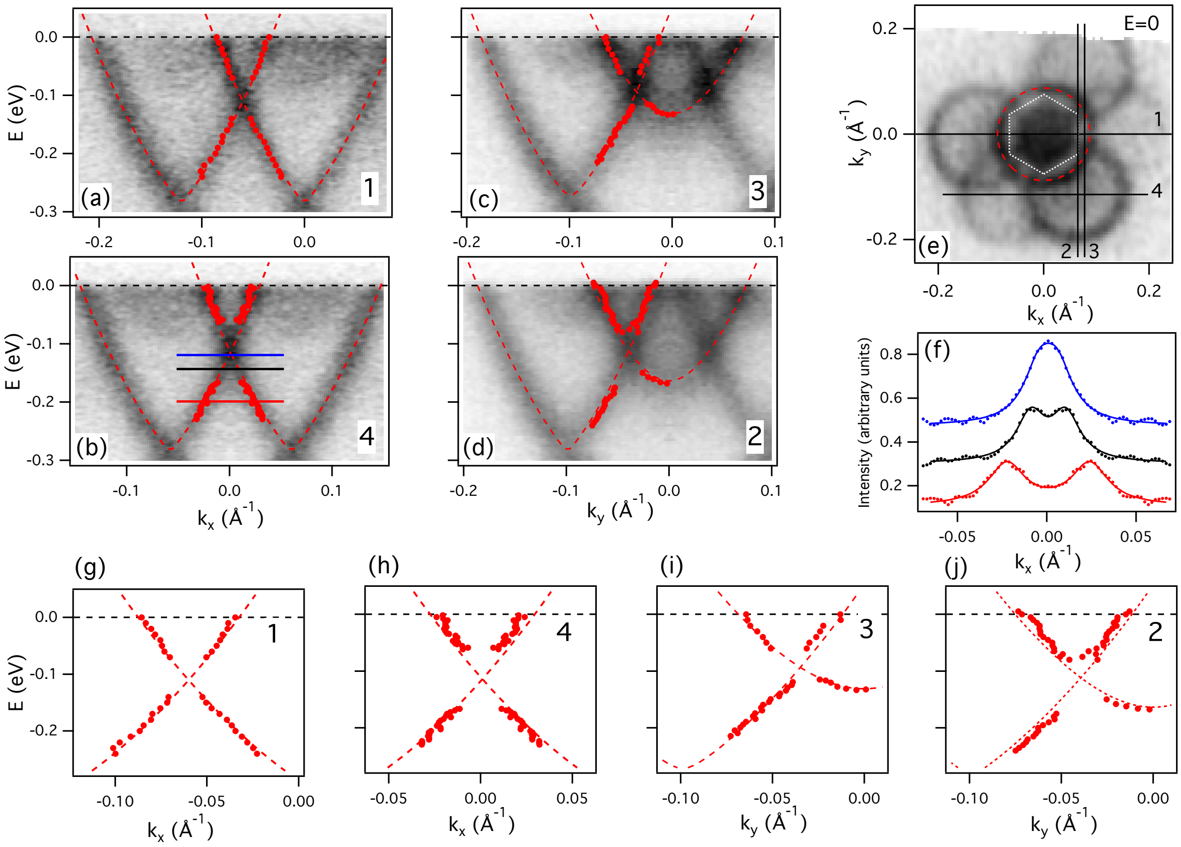

Figure S1: Dispersion of the moiré bands in Xe/Bi_2Se_3. (a–d) Dispersion of the electronic bands along the lines indicated in (e). The red dashed curves represent the original TSS cone and its replicas shifted by the reciprocal wave-vectors of moiré superlattice. The red symbols represent the measured MDC-derived dispersions. (e) Fermi surface with the momentum lines probed in (a–d) as indicated. (f) Several MDCs (points) from the spectrum in (b) at energies as indicated by colored lines, along with the 2-peak Lorentzian fits (lines). The fitting returns $k_x = -0.0224 \pm 0.0003 Å^{-1}$ and $-0.0089 \pm 0.0003 Å^{-1}$ for the left red and black spectrum, respectively. The right counterparts are at $0.0240 \pm 0.0003 Å^{-1}$ and $0.0106 \pm 0.0003 Å^{-1}$, respectively. Small uncertainty in peak position illustrates robustness of the measured dispersions. Closer to the crossing point, fitting becomes less reliable (blue spectrum) and is excluded from the analysis. (g–j) The same as in (a–d), but without the measured photoemission intensity.

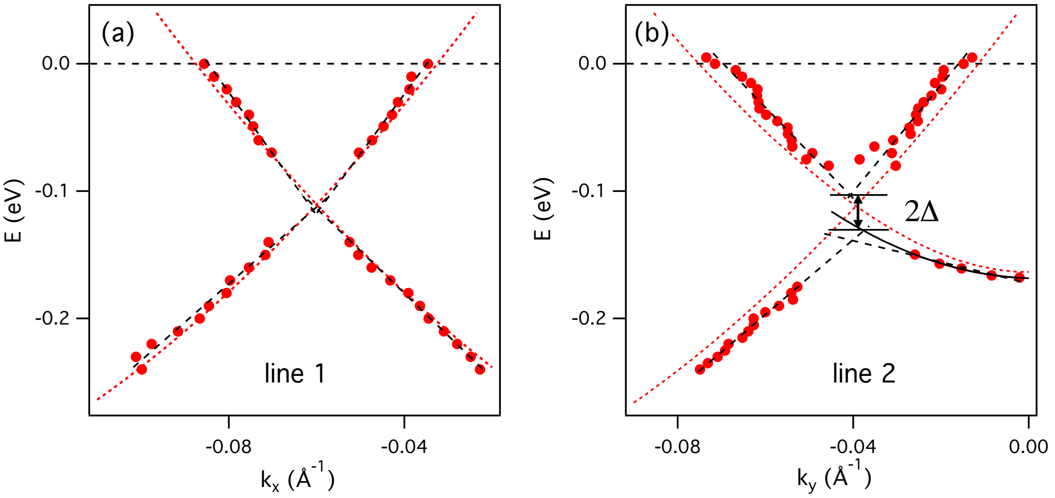

Figure S2: Determination of the moiré hybridization gaps. (a-b) Zoomed-in dispersions along the momentum lines 1 and 2 from Fig. S1, corresponding to the crossings at M' and K', respectively. Red dashed curves and circles correspond to the non-interacting and measured dispersions, respectively. The black dashed lines are linear fits to the measured MDC-derived dispersions. The black solid curve in (b) represents the parabolic fit the the measured EDC-derived dispersion of the main TSS cone. The fitted lines and the parabola are extrapolated to the point of intersect with the counterpart. The energy distance between the upper and lower intersects, if exists, is taken as the moiré gap, 2Δ. The extrapolation of 2nd order polynomial to the intersect generally gives slightly reduced gap magnitude than the linear approximation.

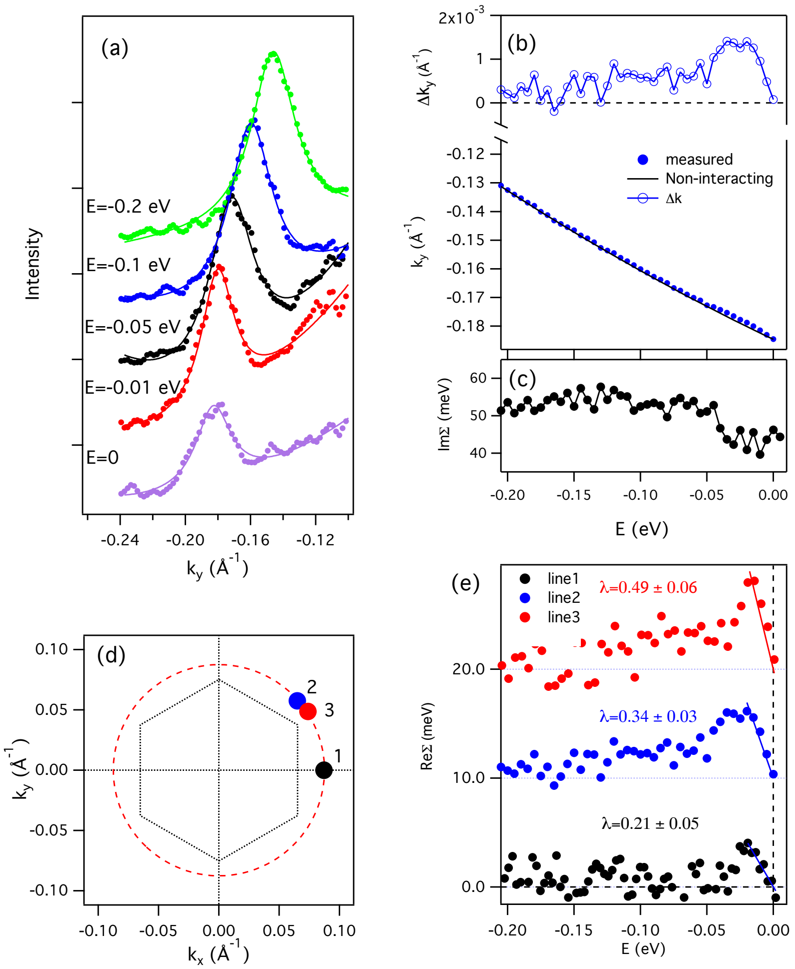

Figure S3: Determination of EPC in Xe/Bi_2Se_3 moiré. (a) Several MDCs from the spectrum taken along the line 2 (Fig. S1(d)). The standard deviation in the peak position is of the order of $\pm 1\times 10^{-4}$ Å^{-1}. (b) Peak positions from MDCs like those from (a) (solid blue circles), plotted along with the non-interacting dispersion (black curve), determined as described in the S.I. text. The top part (open circles) represents the difference in momentum between the two. (c) $\mathrm{Im}\Sigma(E)$ obtained from the width (W) of the MDC peaks, $2\mathrm{Im}\Sigma(E) = W(E)v(E)$, where $v(E)$ is the velocity of non-interacting band shown in (b). (d) Positions on the Fermi surface of the points where the EPC was determined (corresponding to the lines 1-3 from Fig. S1). (e) $\mathrm{Re}\Sigma(E)$ for the three points on the Fermi surface indicated in (d), shifted vertically by a constant offset (10 meV) for clarity. $\mathrm{Re}\Sigma$ is obtained as $\mathrm{Re}\Sigma(E) = \Delta k(E)v(E)$. The lines in (d) are linear fits of $\mathrm{Re}\Sigma(E)$ over the range -20 meV $< E < 0$, whose slope determines λ.
